# Knowledge, Attitudes and Practices on Rabies among Human and Animal Health Professionals in Senegal

**DOI:** 10.3390/pathogens10101282

**Published:** 2021-10-05

**Authors:** Mouhamadou Faly Ba, Ndèye Mbacké Kane, Mamadou Kindi Korka Diallo, Oumar Bassoum, Oumy Kaltome Boh, Fatoumata Zahra Mohamed Mboup, El Hadji Bilal Faye, Andre Pouwedeou Bedekelabou, Sara Danièle Dieng, Fatimata Niang Diop, Médoune Badiane, Valéry Ridde, Adama Faye

**Affiliations:** 1 Institute of Health and Development, Cheikh Anta Diop University, Dakar-Fann BP 5005, Senegal; mamdi45@gmail.com (M.K.K.D.); oumar.bassoum@ucad.edu.sn (O.B.); adama.faye@ucad.edu.sn (A.F.); 2 Ministry of Health and Social Action, Fann Résidence, Rue Aimé Césaire, Dakar BP 4024, Senegal; mbackekane2007@yahoo.fr (N.M.K.); oumyboh@hotmail.com (O.K.B.); zahramboup@yahoo.fr (F.Z.M.M.); 3 Institute of Research for Development, Dakar-Hann BP 1386, Senegal; bilalfaye@hotmail.com (E.H.B.F.); valery.ridde@ird.fr (V.R.); 4 Inter-State School of Veterinary Sciences and Medicine, Cheikh Anta Diop University, Dakar-Fann BP 5005, Senegal; apouwedeo@afrohun.org; 5 Inter-State School of Veterinary Sciences and Medicine, Africa One Health University Network, Dakar-Fann BP 5077, Senegal; sdieng@afrohun.org; 6 Institute of Environmental Sciences, Cheikh Anta Diop University, Dakar-Fann BP 5005, Senegal; fatimata7.niang@ucad.edu.sn; 7 Direction of Veterinary Services, Ministry of Livestock and Animal Productions, Diamniadio, Dakar-Fann BP 45677, Senegal; mbadianevet@gmail.com

**Keywords:** knowledge, attitude, practice, rabies, human health, animal health, one health, zoonosis, Senegal

## Abstract

Rabies is still a public health problem in Senegal. This study aimed to assess the knowledge, attitudes and practices on rabies among human and animal health professionals. It was a cross-sectional, descriptive and analytical study conducted in the Kaffrine district. Data were collected from 28 June to 01 July 2021. An exhaustive recruitment was done, and the final sample size was 95 health professionals. R software was used for descriptive, bivariate and multivariate analyses. Health professionals with sufficient knowledge, positive attitudes and good practices in relation to rabies represented 35.8%, 26.3% and 45.3% of the study respectively. The results of the multivariate analysis showed that professionals who worked in urban areas (AOR = 11.10; 95% CI = [3.50–41.69]) and who worked in animal health (AOR = 7.45; 95% CI = [1.16–70.40]) were more likely to have sufficient knowledge about rabies. Professionals with tertiary education (AOR = 12.40; CI95% = [1.80–268.00]) and with sufficient knowledge (AOR = 3.41; CI95% = [1.01–12.70]) were more likely to have a positive attitude about rabies. Professionals with a positive attitude about rabies (AOR = 3.23; 95% CI = [1.08–10.70]) were more likely to have a good practice when presented with an animal bite case. These results suggest that improving health professionals’ knowledge about rabies is essential in order to influence their attitudes and practices against rabies.

## 1. Introduction

Rabies is considered to be one of the oldest infectious diseases affecting mammals [1]. The disease is caused by a *rhabdovirus* and is usually transmitted to humans through the bite of a rabid animal [2]. It is a major zoonotic disease that threatens global public health [3]. It causes an estimated 59,000 human deaths per year, affecting over 150 countries [4], with Asia being the most affected region, followed by Africa [5,6,7].

In 2020, the number of bite cases reported by the Senegalese Ministry of Health and Social Action was estimated at 3729 cases [8]. Since 2009, an estimated 43 human deaths attributable to the disease have been reported in Senegal [9]. In the Kaffrine region, rabies remains a concern according to epidemiological surveillance data [10]. This region has recorded 400 cases of exposure to a rabies risk, of which 60 were confirmed in 2020 [8]. These figures are far from reflecting reality because, in general, the victims do not come to the health facilities every time; this may be because they underestimate the risk, or because they are unaware of what to do in the event of a bite, or because they anticipate that they will not be able to pay the fees charged at the health facilities. Between 2018 and 2019, three fatal cases were recorded in the region’s health district, despite the existence of effective post-exposure prophylaxis (PEP) [8,11]. As such, the World Health Organization (WHO) guidelines on rabies PEP recommend three important aspects of treatment immediately after exposure to rabid animals: profuse washing of the bite wound with soap and water or detergent, or with water only; administration of the rabies vaccine; and infiltration of rabies immunoglobulin in and around the wound [11,12]. Furthermore, with integrated synergy within the "One Health" framework, rabies can be effectively addressed through integrated animal bite management and PEP, mass vaccination of dogs and community-based rabies awareness programs [13,14,15,16]. In the rabies control policy in Senegal, human and animal rabies is considered to be a notifiable disease. However, it is not free of charge in health care facilities. The costs of a full PEP schedule (excluding RIG) range from 40 to 60 euros and, according to the World Development Indicators, the average monthly disposable salary is around 160 euros [17]. In addition to the direct expenditure on PEP, costs from travel to distant PEP centers and lost income whilst seeking PEP have been reported in studies conducted in other rabies-endemic countries [17].

Knowledge, attitude and practice (KAP) surveys are widely used in health promotion. They are based on the premise that knowledge will improve attitudes and attitudes will improve disease care practices [18] without forgetting the complexity of these processes and thinking about the linearity of decisions. Indeed, gaps in knowledge, attitudes and practices regarding rabies among health professionals are some of the emerging factors attributed to the failure to control and prevent it [5]. For example, in Tanzania, studies have found that veterinary staff and doctors had low knowledge of zoonotic diseases, including rabies [19]. Similarly, in Uganda, research has shown that 56.0% and 75.0% of human and animal health professionals respectively had poor knowledge of rabies and a negative attitude towards rabies management [5]. Collaborative studies have shown that the knowledge and practices of practicing physicians regarding rabies prophylaxis are seriously lacking, particularly in the management of suspected cases of rabies in humans [20,21]. In Senegal, a study conducted in the health district of Sokone showed that only 5.3% of health care providers had a good knowledge of rabies [22]. However, the latter, which is the only study found in the literature involving health professionals, does not include animal health professionals which would allow for a more holistic assessment. KAP studies can be used to organize public health awareness campaigns and provide baseline data for planning, implementing and evaluating national disease control programs [23]. They are also very important for identifying the level of commitment of national disease control programs.

Thus, this study aimed to assess the KAP on rabies among human and animal health professionals in order to strengthen rabies control and elimination strategies. The results of this study could be used to implement rabies training programs in Kaffrine and other high exposure areas to improve rabies control and management of animal and human bites. The results will serve as indicators to strengthen existing policies and actions and provide a baseline for future evaluation of the national rabies elimination program in Senegal and other countries of similar socio-economic status by 2030.

## 2. Materials and Methods

### 2.1. Study Area

The Kaffrine district belongs to the eponymous region located in central Senegal. It covers the department of the same name with an area of 2779 km2. In terms of health infrastructure, the Kaffrine district includes a Public Health Establishment (Level 2), one health center, thirty-two health posts, forty-two health huts, five private pharmacies, one Regional Service for Livestock and Animal Production, a Departmental Service for Livestock and Animal Production, seven veterinary posts and two private veterinary clinics. In the human health facilities in the district, human rabies immune globulin and rabies vaccine are not widely available and the cost of these are very expensive. The animal health services are mainly involved in the annual mass vaccination campaigns on World Rabies Day by inviting dog owners to come to the services and to designated stands outside the service. In terms of human resources, the district’s staff is comprised of 5 doctors (2 of whom work in the emergency department of the Kaffrine Regional Hospital), 2 senior technicians, 34 state midwives, 28 state nurses, 21 assistant nurses, a preventionist, 4 hygiene officers, 18 drivers (4 of whom work at the health center), 57 matrons, 38 community health workers, 1 secretary, 1 manager, 328 relays, 112 Bajenu Gox, 4 veterinarians, 4 livestock technical officers, and 1 livestock technical engineer.

### 2.2. Type, Period and Study Population

This was a cross-sectional, descriptive and analytical study. The data were collected from 28 June to 1 July 2021. The study population consisted of human and animal health professionals practicing in the Kaffrine district.

### 2.3. Sampling

The following inclusion criteria were set for respondents from both categories of professionals who freely consented to participate in the study:

- An animal health professional present and working in the Kaffrine district at the time of the survey in the veterinary work station visited.
- A human health professional qualified as a nurse, nursing assistant, doctor or pharmacist, present and working in the Kaffrine district at the time of the survey in the health facility visited.

An exhaustive recruitment of human and animal health professionals in the Public Health Establishment, the health center, the health posts and the veterinary services was done. A total of 102 human and animal health professionals were eligible for the survey.

### 2.4. Data Collection

A questionnaire for health professionals was used to collect information based on different appropriate conceptual frameworks. A pre-test was carried out to ensure that the questions were clear and understandable. The questionnaire consisted of the following four dimensions:

- Socio-demographic characteristics:

Variables, such as place of work, gender, age, marital status, education level, job title, years in service and pet ownership, were collected.

- Knowledge:

A method described by Koruk et al. [24] was used to measure health professionals’ knowledge of rabies using 16 questions. A holistic approach (from cause of infection to management) to rabies was important in this study. The questions asked about rabies were as follows: knowledge of the disease, its cause, main reservoirs, species affected, mode of transmission, groups of people most at risk, incubation period in animals, signs/symptoms in animals, period of contagiousness in dogs/cats, incubation period in humans, signs/symptoms in humans, preventive measures, first aid given to a patient that has been bitten/scratched by a suspected rabid animal, prevention of rabies after an animal bite, vaccination schedule/scheme for pets and vaccination schedule/scheme for humans.

For scoring purposes, these were weighted in the same way. Each correct answer was worth 1 point and other answers were worth 0 points. Knowledge of rabies was defined as a binary variable (sufficient vs. insufficient). In accordance with Monje et al. [5], The health provider was considered to have sufficient knowledge when the sum of the scores of the 16 questions was greater than the mean score.

- Attitudes:

Ten questions made up the attitude section [5]. They were composed using a 5-point Likert scale (5: strongly agree to 1: strongly disagree). These questions included: do you think rabies is not caused by bacteria; do you think rabies affects mammals; not all domestic animals are the only sources of rabies infection; do you think bats transmit rabies; do you think rabies can be transmitted by aerosols; do you advise a person to seek treatment in a health facility; the willingness of communities to vaccinate their pets; do you think that vaccination of pets contributes greatly to rabies control in Kaffrine district; would awareness raising efforts against rabies lead to rabies control; and is there a need for joint efforts of the medical and veterinary sectors to control rabies.

For each question, "strongly agree" and "agree" were scored as 1 point and the others as 0 points. Attitude was defined as a binary variable (positive vs. negative). As suggested in two studies [5,12], professionals who had more than 86% of the score (9 or 10) were considered to have a positive attitude.

- Practices:

This section of the questionnaire initially included questions that concerned human health professionals only regarding the management of animal bites: wash the wound(s) quickly with soap and water, a detergent and then rinse thoroughly with pure water for at least 15 min, then disinfect by applying an antiseptic solution, prevent tetanus, assess the risk of rabies infection, categorize the case, start post-exposure prophylaxis if necessary and notify by filling in a declaration form. Following these questions, there were questions that concerned animal health professionals only and related to the management of animal bites: observation of the dog for 15 days, referral of the patient to the doctor or the head nurse, washing the wound with soap and water for 15 min, slaughtering the biting animal, slaughtering the bitten animal, vaccination of the bitten animal, informing the administrative authority and notifying the case.

Specific questions that regarded the management of a bite case were measured by a 5-point Likert scale (5: strongly agree to 1: strongly disagree). This variable was converted into a binary score (good vs. bad). Human and animal health professionals were considered to have good practice in dealing with an animal bite when all responses were consistent with the existing literature on rabies prevention and control [11,25].

Five investigators went into health care facilities to recruit health professionals. They collected data through individual face-to-face interviews. The facilities were classified into different axes according to their geographical location in order to have a good progression. Tablets equipped with Kobotoolbox software [26] were used to administer the questionnaire. Data quality control was carried out by training the interviewers, pre-testing the tools, scanning, collecting the data on a tablet and monitoring the data collection in real time on a daily basis.

### 2.5. Data Analysis

The data were analyzed using R software version 4.0.5. A descriptive analysis was carried out on all the data collected. The quantitative variables were described through the mean with its standard deviation and the qualitative variables through the absolute and relative frequencies. In the bivariate analysis, the Chi2 test and Fisher’s exact test were performed according to the conditions of applicability. These tests were used to search for pairwise associations with an alpha risk of 5%. The following comparisons were made:

- Knowledge (binary variable) as dependent variable and socio-demographic characteristics as independent variable;
- Attitude (binary variable) as dependent variable and socio-demographic characteristics plus knowledge as independent variable;
- Practice (binary variable) as dependent variable and socio-demographic characteristics plus attitude and knowledge as independent variable.

To control for confounding factors, binomial logistic regression was performed. All variables used in the bivariate analysis were included in our models. The fit of the model was measured using the Hosmer-Lemeshow test [27]. This multivariate analysis was used to determine the adjusted odds ratios (AOR) and their confidence intervals.

## 3. Results

### 3.1. Descriptive Analysis

#### 3.1.1. Participation Rate

A total of 95 health professionals participated in the study, representing a participation rate of 93.1%. The remaining seven were not present at the time of the survey.

#### 3.1.2. Socio-Demographic Characteristics

Of the health professionals surveyed, 46.3% were men. Those living in urban areas accounted for 34.7%. The average age was 36.1 ± 8.6 years and 47.4% were over 35 years old. The professionals had been in service for 8.8 ± 6.7 years. In addition, 41.1% of the professionals owned a pet and 7.1% had undergone rabies refresher training (Table 1).

#### 3.1.3. Knowledge

In the survey, 85.9% of the professionals said they knew about rabies. The rest of the professionals were not sure if they knew about the virus. Professionals generally had good knowledge of the pathogen that causes rabies, the species that rabies affects, the modes of transmission of rabies and the first aid given to patients after a bite from a suspected rabid animal, with proportions above 60.0%. On the other hand, the knowledge of professionals was low on questions related to the groups of people most exposed to rabies and on the vaccination schedule for animals and humans against rabies (Table A1).

The average score of the professionals’ knowledge of rabies was 7.1 ± 1.9. The score ranged from 2 to 12. Table 2 shows the distribution of professionals according to their knowledge of rabies and level of practice. The knowledge score ranged from 2 to 11 among human health professionals, while it ranged from 7 to 12 among animal health professionals. Professionals with sufficient knowledge of rabies represented 35.8% of the study.

#### 3.1.4. Attitude

Professionals who strongly agreed or agreed that rabies was not caused by bacteria represented 51.6%. In this sense, the level of agreement of professionals was higher than 50.0% for seven statements (Table A2). 

The mean score of the professionals’ attitude towards rabies was 7.7 ± 1.1. The score ranged from 5 to 10. Table 2 shows the distribution of professionals according to attitude on rabies and level of practice. The knowledge score ranged from 5 to 10 for human health professionals, while it ranged from 7 to 10 for animal health professionals. Professionals with a positive attitude towards rabies represented 26.3% of the study.

#### 3.1.5. Practice

In the study, 27.4% of the professionals had employed a jointly collaborative effort between medical and veterinary services when dealing with a suspected rabies case. For human health professionals, the only question on aspects related to the management of suspected rabies cases below 60.0% agreement was the one related to management by disinfection using an antiseptic solution (56.8%) (Table A3). For the questions aimed at animal health professionals, the proportions of professionals who strongly agreed/agreed on placing the dog under observation for 15 days and referring the patient to the doctor or the head nurse were 100.0% and 89.9% respectively. The majority of animal health professionals disagreed or strongly disagreed with the vaccination of the biting animal and the vaccination of the bitten animal (87.5% and 75.0%) (Table A3). The good practice of the professionals in front of an animal bite case was estimated at 45.3%.

### 3.2. Bivariate Analysis

The proportion of professionals practicing in urban areas who had sufficient knowledge about rabies was 60.6%, while the rate was 22.6% among those practicing in rural areas (*p* = 0.001). The proportion of professionals with tertiary education who had a positive attitude about rabies was 32.4%, while it was 4.8% among those with secondary education (*p* = 0.024). The proportion of professionals with less than 10 years of service who had good practice in dealing with animal bites was 51.8%. This proportion was 35.9% among professionals with experience of 10 years or more (*p* = 0.187) (Table 3).

### 3.3. Multivariate Analysis

The aim was to model sufficient knowledge about rabies, positive attitudes about rabies and good practice when faced with a bite from a suspected rabid animal by showing the factors associated with these in a consistent way. The Hosmer-Lemeshow tests (*p* = 0.06; *p* = 0.98; *p* = 0.81) showed a good fit of our three models. The results of the multivariate analysis showed that professionals who worked in urban areas (ORA = 11.10; CI95% = [3.50–41.69]) and who worked in animal health (ORA = 7.45; CI95% = [1.16–70.40]) were more likely to have sufficient knowledge about rabies. Professionals with a tertiary education (AOR = 12.40; CI95% = [1.80–268.00]) and with sufficient knowledge (AOR = 3.41; CI95% = [1.01–12.70]) were more likely to have a positive attitude about rabies. Professionals with a positive attitude about rabies (AOR = 3.23; 95% CI = [1.08–10.70]) were more likely to have a good practice in front of an animal bite case (Table 4).

## 4. Discussion

Rabies is a public health problem, mainly in Asia and Africa. ‘United against rabies’ collaboration through the ‘One Health’ approach is the current motto of the global rabies elimination strategy [28]. Our study provides a picture of the current knowledge, attitudes and practices of human and animal health professionals on rabies. It also shows the importance of intersectoral collaboration and the benefit of developing an animal health system.

The results of the study showed that more than half of the human health professionals surveyed (67.8%) had insufficient knowledge about rabies. This result is similar to those obtained in Uganda, Turkey, Chad and Senegal [5,22,24,29]. In contrast, studies in Vietnam and the United States showed moderate and high levels of knowledge among human health professionals, respectively, despite gaps in some areas [30,31]. These results may be explained by the lack of ongoing training of professionals on rabies in Senegal. In effect, only 7.4% of professionals had previously attended a rabies refresher course or workshop. This is a very poor result and should encourage the development of training for these human health professionals. Human health professionals are generally aware of the risk of rabies, but their knowledge of rabies exposure management and prevention often needs updating [32]. Health authorities should provide more detailed information to these professionals and to the general population about rabies risk. Well-trained health professionals are essential for increasing access to PEP by improving compliance. In contrast to human health professionals, 75.0% of animal health professionals surveyed had sufficient knowledge about rabies. Similar results were found in Uganda and the USA [5,30]. This result is in contrast to those obtained in Tanzania and Chad, where animal health workers had poor knowledge of rabies [19,29]. The results of the multivariate analysis showed that animal health workers were 7.45 times more likely to have sufficient knowledge about rabies than human health workers. In this regard, studies conducted in the United States and Australia have shown that, overall, animal health professionals were more knowledgeable about rabies than human health professionals [30,33,34]. This could be explained by the fact that veterinarians conduct ongoing zoonotic disease risk assessments as part of their daily professional practice, whereas for human health professionals this is a small component of their clinical practice. The results call for a rapprochement of the two health systems and greater intersectoral collaboration in the ‘One Health’ perspective. The multivariate analysis also showed that health professionals working in urban areas were 11.10 times more likely to have sufficient knowledge about rabies than those working in rural areas. This result is identical to that obtained in Bangladesh [28]. A study by Alam et al. [35] concluded that adequate knowledge of rabies was strongly correlated with people living in urban areas due to the ease of education and higher standard of living.

The study showed that the majority of health professionals had a negative attitude towards rabies management. These results were consistent with related studies in Uganda and Kenya [5,36]. The latter study recommended that public health workers need more knowledge, correct attitudes and appropriate skills to enable them to carry out surveillance and teach the public about zoonotic disease control measures. In the study, attitude was influenced by the level of education. Health professionals with a tertiary education were 12.40 times more likely to have a positive attitude. This is similar to the result found in Uganda [5].

Less than half of health professionals (45.3%) had good practice in dealing with a suspected animal bite. The WHO recommends immediate initiation of post-exposure prophylaxis with careful wound cleansing, application of local treatment, administration of a series of doses of a potent and effective standard rabies vaccine and administration of rabies immunoglobulin as indicated [11]. Post-exposure prophylaxis is important because of its ability to prevent the progression of rabies virus to the nerves [37]. It is also important to work in synergy with veterinary services for a more comprehensive management. In the study, 27.4% of professionals employed a joint effort between medical and veterinary services when dealing with a suspected rabies case. This shows the progress that needs to be made to operationalize the ‘One Health’ approach. Collaboration may also be hampered by time constraints, a lack of understanding of the health benefits, and few established relationships between practitioner groups [33]. Current good practice in rabies control is to adopt the ‘One Health’ approach, in which animal and human health professionals and other key stakeholders work together in community awareness and animal vaccination campaigns led by the Direction of Veterinary Services [5]. This approach focuses on collaborative efforts that harness and coordinate the power of multidisciplinary and cross-sectoral teams and resources to be applied locally, nationally and internationally for optimal human, animal and environmental health [38]. The common theme in the application of the ‘One Health’ approach to rabies management is collaboration across disciplines and sectors.

This study showed that health professionals’ knowledge of rabies influenced their attitudes about rabies, which in turn influenced their practice in the management of a suspected rabies case. These results are consistent with those found in Uganda [5] and the findings of Mascie-Taylor et al. [18]. It should also be noted that our study showed that the level of education played a significant role in the level of knowledge about rabies [5]. These results suggest that there is a need to improve health professionals’ knowledge of rabies in order to influence their attitudes and practices against rabies.

This study has some limitations. Doctors in medical specialties were not targeted, even though they are likely to encounter patients potentially exposed to rabies. The study was conducted in only one health district out of the 79 in Senegal. The results cannot therefore be generalized to the whole country. However, it can be said that the health districts are organized in the same way and the professionals trained in the same schools with a certain homogeneity [39]. In addition, the sample of animal health professionals was low. Despite these limitations, this survey provided useful data to guide public health efforts in rabies control in the health districts. In addition, the gaps identified in this study will be further used to develop targeted joint educational interventions to build capacity in health professionals and explore avenues to facilitate inter-professional relationships that will foster collaboration and guidance with the ultimate goal of improving human and animal health outcomes.

## 5. Conclusions

Health professionals play a vital role in primary health care and disease surveillance in humans and animals. This study has shown that there are gaps in knowledge, attitudes and practices towards rabies in one district in Senegal. Human and animal health professionals have different but complementary knowledge and skills, with the potential to improve the clinical management of zoonotic diseases in human and animal patients by adopting a ‘One Health’ approach that promotes interprofessional collaboration. Medical education on life-threatening infections such as rabies, including available preventive and prophylactic interventions, is necessary and should be ongoing. In addition, qualitative research may be needed to better understand the results and to analyze systems in more detail.

## Figures and Tables

**Table 1:** Distribution of health professionals by socio-demographic characteristics (N = 95).

	N (%)
**Exercise Area**	
Rural	62 (65.3%)
Urban	33 (34.7%)
**Sex**	
Woman	51 (53.7%)
Male	44 (46.3%)
**Age**	
≤35 years	50 (52.6%)
>35 years	45 (47.4%)
**Marital Status**	
Unmarried	21 (22.1%)
Married	74 (77.9%)
**Education Level**	
Secondary	21 (22.1%)
Tertiary	74 (77.9%)
**Type of Qualification**	
Human health professional	87 (91.6%)
Animal health professional	8 (8.4%)
**Year in Service**	
<10 Years	56 (58.9%)
≥10 Years	39 (41.1%)
**Possession of Pets**	
No	56 (58.9%)
Yes	39 (41.1%)
**Previously Attended Rabies Training or Refresher Courses**	
No	88 (92.6%)
Yes	7 (7.4%)

**Table 2:** Distribution of professionals according to knowledge and attitude about rabies and level of practice.

	Human Health Professional	Animal Health Professional	All
	N = 87	N = 8	N = 95
**Rabies Knowledge Score**			
2	1 (1.1%)	0 (0.0%)	1 (1.1%)
3	3 (3.4%)	0 (0.0%)	3 (3.2%)
4	5 (5.7%)	0 (0.0%)	5 (5.3%)
5	10 (11.5%)	0 (0.0%)	10 (10.5%)
6	11 (12.6%)	0 (0.0%)	11 (11.6%)
7	29 (33.3%)	2 (25.0%)	31 (32.6%)
8	14 (16.1%)	0 (0.0%)	14 (14.7%)
9	8 (9.2%)	4 (50.0%)	12 (12.6%)
10	3 (3.4%)	0 (0.0%)	3 (3.2%)
11	3 (3.4%)	1 (12.5%)	4 (4.2%)
12	0 (0.0%)	1 (12.5%)	1 (1.1%)
**Rabies Attitude Score**			
5	2 (2.3%)	0 (0.0%)	2 (2.1%)
6	14 (16.1%)	0 (0.0%)	14 (14.7%)
7	20 (23.0%)	3 (37.5%)	23 (24.2%)
8	31 (35.6%)	0 (0.0%)	31 (32.6%)
9	18 (20.7%)	4 (50.0%)	22 (23.2%)
10	2 (2.3%)	1 (12.5%)	3 (3.2%)

**Table 3:** Distribution of professionals according to their KAP about rabies and socio-demographic characteristics.

	Sufficient Knowledge	*p*-Value	Positive Attitude	*p*-Value	Good Practice	*p*-Value
	N (%)		N (%)		N (%)	
**Location**		**0.001**		0.062		0.807
Rural	62 (22.6%)		62 (19.4%)		62 (43.5%)	
Urban	33 (60.6%)		33 (39.4%)		33 (48.5%)	
**Sex**		0.747		0.614		1.000
Woman	51 (33.3%)		51 (29.4%)		51 (45.1%)	
Male	44 (38.6%)		44 (22.7%)		44 (45.5%)	
**Age**		0.550		0.873		0.236
≤35 years	50 (32.0%)		50 (28.0%)		50 (52.0%)	
>35 years	45 (40.0%)		45 (24.4%)		45 (37.8%)	
**Education Level**		0.612		**0.024**		1.000
Secondary	21 (42.9%)		21 (4.8%)		21 (47.6%)	
Tertiary	74 (33.8%)		74 (32.4%)		74 (44.6%)	
**Type of Qualification**		**0.023**		**0.028**		0.725
Human health professional	87 (32.2%)		87 (23.0%)		87 (46.0%)	
Animal health professional	8 (75.0%)		8 (62.5%)		8 (37.5%)	
**Year in Service**		0.502		0.558		0.187
<10 years	56 (32.1%)		56 (23.2%)		56 (51.8%)	
≥10 years	39 (41.0%)		39 (30.8%)		39 (35.9%)	
**Possession of Pets**		0.814		1.000		0.723
No	56 (33.9%)		56 (26.8%)		56 (42.9%)	
Yes	39 (38.5%)		39 (25.6%)		39 (48.7%)	
**Followed a Rabies Training or Refresher Course in the Past**		0.698		0.375		0.123
No	88 (35.2%)		88 (25.0%)		88 (47.7%)	
Yes	7 (42.9%)		7 (42.9%)		7 (14.3%)	
**Knowledge**		−		**0.027**		0.962
Insufficient	−		61 (18.0%)		61 (44.3%)	
Sufficient	−		34 (41.2%)		34 (47.1%)	
**Attitude**		−		−		0.136
Negative	−		−		70 (40.0%)	
Positive	−		−		25 (60.0%)	

Bold if *p* < 0.05.

**Table 4:** Results of the multivariate analysis.

Characteristics	Knowledge Model			Attitude Model			Practice		
	AOR ^1^	95% CI ^1^	*p*-Value	AOR ^1^	95% CI ^1^	*p*-Value	AOR ^1^	95% CI ^1^	*p*-Value
**Location**			**<0.001**			0.299			0.701
Rural	**1.00**	**−**		1.00	−		1.00	−	
Urban	**11.10**	**3.50, 41.69**		2.03	0.55, 7.40		1.27	0.42, 3.90	
**Sex**			0.499			0.067			0.600
Woman	1.00	−		1.00	−		1.00	−	
Male	0.67	0.21, 2.00		0.30	0.07, 1.09		1.28	0.48, 3.46	
**Age**			0.799			0.299			0.599
≤35 years	1.00	−		1.00	−		1.00	−	
>35 years	0.86	0.25, 2.82		0.50	0.12, 1.80		0.78	0.27, 2.23	
**Education Level**			**0.021**			**0.007**			0.500
Secondary	**1.00**	**−**		**1.00**	**−**		1.00	−	
Tertiary	**0.24**	**0.07, 0.81**		**12.40**	**1.80, 268.00**		0.69	0.22, 2.15	
**Type of qualification**			**0.034**			0.139			0.601
Human health professional	**1.00**	**−**		1.00	−		1.00	−	
Animal health professional	**7.45**	**1.16, 70.40**		4.01	0.63, 31.1		0.65	0.09, 4.09	
**Year in Service**			0.899			0.800			0.301
<10 years	1.00	−		1.00	−		1.00	−	
≥10 years	1.09	0.33, 3.61		1.22	0.33, 4.69		0.59	0.20, 1.70	
**Possession of Pets**			0.199			0.599			0.501
No	1.00	−		1.00	−		1.00	−	
Yes	2.17	0.69, 7.23		1.47	0.41, 5.31		1.39	0.51, 3.81	
**Followed a Rabies Training or Refresher Course in the Past**			0.499			0.500			0.101
No	1.00	−		1.00	−		1.00	−	
Yes	1.86	0.25, 12.2		2.08	0.25, 18.5		0.19	0.01, 1.33	
**Knowledge**						**0.048**			0.799
Insufficient				**1.00**	**−**		1.00	−	
Sufficient				**3.41**	**1.01, 12.70**		0.89	0.31, 2.55	
**Attitude**									**0.036**
Negative							**1.00**	**−**	
Positive							**3.23**	**1.08, 10.70**	

^1^ AOR = Adjusted Odds Ratio, CI = Confidence Interval. Bold if *p* < 0.05.

## Data Availability

Data are available upon request to the authors.

## Appendix A

**Table A1:** Distribution of professionals according to rabies knowledge characteristics (N = 95).

**Characteristics**	N (%)
**Do You Know Rabies?** Yes	85 (89.5%)
**What Type of Pathogen Causes Rabies?**	
Virus *	66 (69.5%)
Bacteria	16 (16.8%)
Mushrooms	3 (3.2%)
Pests	5 (5.3%)
Don’t know	5 (5.3%)
**What Are the Main Reservoirs of Rabies?**	
Many wild and domestic canids and other mammals	51 (53.7%)
All animals	18 (18.9%)
Only domestic canines	14 (14.7%)
Only wild canids *	12 (12.6%)
**What Species Does Rabies Affect?**	
All mammals *	81 (85.3%)
Humans only	6 (6.3%)
Dogs only	7 (7.4%)
Other	1 (1.1%)
**How Is Rabies Transmitted? ^†^**	
Bite from an infected animal *	94 (98.9%)
Scratch of an infected animal *	70 (73.7%)
Contact with the animal’s skin	7 (7.4%)
Licking on a wound by an infected animal *	69 (72.6%)
Wizard	2 (2.1%)
**Which Groups of People Are most Prone to Animal Bites?**	
Children *	38 (40.0%)
Young people	4 (4.2%)
Adults	2 (2.1%)
All groups	51 (53.7%)
**What Is the Incubation Period for Rabies in Animals?**	
1 to 3 days	10 (10.5%)
10 days to 2 months or more *	45 (47.4%)
25 days to 150 days or more (5 months)	8 (8.4%)
Other	2 (2.1%)
Don’t know	30 (31.6%)
**List the Signs of Rabies in Animals? ^†^**	
Change in behavior by hiding in dark corners *	82 (86.3%)
Aggressiveness with a loss of distrust for humans *	89 (93.7%)
Profuse salivation *	81 (85.3%)
Tendency to convulse	55 (57.9%)
Paralysis *	42 (44.2%)
Other	3 (3.2%)
**How Long Is Rabies Contagious in Dogs/Cats?**	
3–7 days *	30 (31.6%)
One month	23 (24.2%)
One year	1 (1.1%)
Other	3 (3.2%)
Don’t know	38 (40.0%)
**What Is the Incubation Period for Rabies in Humans?**	
2–3 months *	23 (24.2%)
3–4 days	38 (40.0%)
9 days to 7 years	4 (4.2%)
Other	7 (7.4%)
Don’t know	23 (24.2%)
**List the Signs of Rabies in Humans? ^†^**	
Fever *	71 (74.7%)
Pain or tingling *	71 (74.7%)
Headache; Dizziness *	71 (74.7%)
Nausea; Vomiting *	47 (49.5%)
Paresthesias *	54 (56.8%)
Delusions	79 (83.2%)
Convulsions	70 (73.7%)
Anxiety *	59 (62.1%)
Hydrophobia *	58 (61.1%)
Aerophobia *	43 (45.3%)
Paralysis *	49 (51.6%)
**What Are the Ways to Prevent Rabies? ^†^**	
Vaccination of pets against rabies *	90 (94.7%)
Raising community awareness about rabies *	91 (95.8%)
Active surveillance of rabies in animals	85 (89.5%)
Detention and 15 days clinical observation for any healthy looking dog or cat known to have bitten a person	91 (95.8%)
Immediately submit intact heads of presumed rabid animals packed in ice to a laboratory	84 (88.4%)
Immediately put down unvaccinated dogs or cats bitten by a known rabid animal	77 (81.1%)
**What First Aid Is Given to a Patient after a Bite/Scratch from a Suspected Rabid Animal?**	
Immediate and thorough cleansing of the wound with soap and water, followed by ethanol or iodine	92 (96.8%)
Suturing the wound	2 (2.1%)
Don’t know	1 (1.1%)
**What Are the Measures for the Prevention of Rabies in Humans after an Animal Bite? ^†^**	
Immediate and thorough cleansing of the wound with soap or detergent and water	92 (96.8%)
Take to the health center for administration of human rabies immunoglobulin as soon as possible	94 (98.9%)
Suturing the wound	10 (10.5%)
**What is the Vaccination Regime/Schedule for Pets against Rabies?**	
Once a year *	35 (36.8%)
Once every 2 or 3 years	4 (4.2%)
Once in a lifetime	5 (5.3%)
Don’t know	51 (53.7%)
**What is the Vaccination Schedule for Humans against Rabies?**	
Once a year	13 (13.7%)
Vaccinate high-risk groups *	24 (25.3%)
Once every 2 years	7 (7.4%)
Once in a lifetime	5 (5.3%)
Other	4 (4.2%)
Don’t know	42 (44.2%)

^†^ Multiple choice; * Right answer.

## Appendix B

**Table A2:** Distribution of professionals according to their attitude to rabies (N = 95).

	Totally Agree	I Agree	Neither Disagree nor Agree	No Agreement	Not at All in Agreement
**Attitude**					
Do you believe that rabies is not caused by bacteria	39 (41.1%)	10 (10.5%)	9 (9.5%)	23 (24.2%)	14 (14.7%)
Do you think rabies affects mammals	67 (70.5%)	20 (21.1%)	0 (0.0%)	6 (6.3%)	2 (2.1%)
Not all pets are the only sources of rabies infection	54 (56.8%)	21 (22.1%)	3 (3.2%)	8 (8.4%)	9 (9.5%)
Do you think bats transmit rabies	24 (25.3%)	17 (17.9%)	22 (23.2%)	16 (16.8%)	16 (16.8%)
Did you know that rabies can be transmitted by aerosols	4 (4.2%)	4 (4.2%)	10 (10.5%)	33 (34.7%)	44 (46.3%)
Would you advise a person bitten/scratched by a suspected rabid animal to seek treatment at a medical facility	94 (98.9%)	1 (1.1%)	0 (0.0%)	0 (0.0%)	0 (0.0%)
Are communities ready to vaccinate their pets?	90 (94.7%)	5 (5.3%)	0 (0.0%)	0 (0.0%)	0 (0.0%)
Do you think that the vaccination of pets contributes greatly to the fight against rabies in the district of Kaffrine	90 (94.7%)	4 (4.2%)	0 (0.0%)	1 (1.1%)	0 (0.0%)
Will awareness efforts lead to effective rabies control in Kaffrine district	86 (86.3%)	11 (11.6%)	1 (1.1%)	1 (1.1%)	0 (0.0%)
Is there a need for human and animal health professionals to work in synergy to control rabies	89 (93.7%)	5 (5.3%)	1 (1.1%)	0 (0.0%)	0 (0.0%)

## Appendix C

**Table A3:** Distribution of professionals according to their practices when faced with a case of a bite from a suspected rabid animal.

Practices	Totally Agree	I Agree	Neither Disagree nor Agree	No Agreement	Not at All in Agreement
**Human Health Professionals (N = 87)**					
Wash the wound(s) quickly with soap and water, detergent and then rinse thoroughly with clean water for at least 15 min	78 (89.7%)	7 (8.0%)	1 (1.1%)	1 (1.1%)	0 (0.0%)
Disinfect by applying an antiseptic solution (70 °C alcohol or Polyvidone iodine)	37 (42.5%)	12 (13.8%)	3 (3.4%)	17 (19.5%)	18 (20.7%)
Preventing tetanus	67 (77.0%)	13 (14.9%)	3 (3.4%)	2 (2.3%)	2 (2.3%)
Assessing the risk of rabies infection	66 (75.9%)	15 (17.2%)	3 (3.4%)	2 (2.3%)	1 (1.1%)
Categorize the type of bite	65 (74.7%)	14 (16.1%)	4 (4.6%)	1 (1.1%)	3 (3.4%)
Start post-exposure prophylaxis if appropriate and according to the chosen protocol recommended by WHO	76 (87.4%)	6 (6.9%)	2 (2.3%)	2 (2.3%)	1 (1.1%)
Report the bite, scratch, lick or any other aggression by a suspect animal by filling in a report form	83 (95.4%)	2 (2.3%)	2 (2.3%)	0 (0.0%)	0 (0.0%)
**Animal Health Professionals (N = 8)**					
Putting the dog under observation for 15 days	7 (87.5%)	1 (12.5%)	0 (0.0%)	0 (0.0%)	0 (0.0%)
Refer the patient to the doctor or the head nurse	7 (87.5%)	1 (12.5%)	0 (0.0%)	0 (0.0%)	0 (0.0%)
Wash the wound with soap and water for 15 min	7 (87.5%)	1 (12.5%)	0 (0.0%)	1 (11.1%)	1 (11.1%)
Killing the biting animal	1 (12.5%)	0 (0.0%)	0 (0.0%)	3 (37.5%)	4 (50.0%)
Killing the bitten animal	3 (37.5%)	0 (0.0%)	0 (0.0%)	1 (12.5%)	4 (50.0%)
Vaccinate the biting animal	0 (0.0%)	0 (0.0%)	1 (12.5%)	3 (37.5%)	4 (50.0%)
Vaccinate the bitten animal	1 (12.5%)	0 (0.0%)	1 (12.5%)	3 (37.5%)	3 (37.5%)
Informing the administrative authority	6 (75.0%)	1 (12.5%)	1 (12.5%)	0 (0.0%)	0 (0.0%)
Notify the case	7 (87.5%)	1 (12.5%)	0 (0.0%)	0 (0.0%)	0 (0.0%)
